# Phagocytic microglia in development: Are they what they eat?

**DOI:** 10.1016/j.bbih.2021.100373

**Published:** 2021-10-20

**Authors:** Jonathan W. VanRyzin

**Affiliations:** Department of Pharmacology, University of Maryland School of Medicine, Baltimore, MD, United States

**Keywords:** Brain development, Behavior, Microglia, Phagocytosis, Trained immunity

## Abstract

Microglia, the innate immune cells of the brain, are indispensable for proper brain development. As professional phagocytes, microglia engulf other cells within distinct developmental niches to sculpt the architecture of the brain. Here, I highlight the age-, brain region-, and substrate-dependent diversity of developmental phagocytosis, and pose the idea that phagocytosis may, in turn, drive changes in microglia phenotype. Ultimately, phagocytosis might be just as important for shaping microglia function as it is for shaping the brain.

## 1 Introduction

Microglia are the resident macrophages of the brain. Over the past few decades, we have come to appreciate the multitude of functions these specialized phagocytes perform. From synaptic pruning to cytokine secretion, and from early development to aging and neurological disease, microglia survey, sculpt, and secrete to ensure that the brain develops and functions appropriately (for review, see Sierra et al., 2019).

As macrophages (from the Greek words meaning “big eaters”), their defining—and perhaps most important—characteristic is their ability to “eat”, or phagocytose, other materials. Traditional views held microglia to be simply the brain's “garbage collectors”, serving only to clean up dead cells, debris, and pathogens. However, we are now beginning to appreciate that phagocytosis—particularly during development—is an essential immune function that has been coopted to sculpt and refine brain circuitry (Mosser et al., 2017; Thion et al., 2018b).

In this review, I will discuss the recent evidence that demonstrates microglial phagocytosis is an essential process that shapes the development of the brain. I will highlight the transient nature of this process and focus on the diversity of endpoints that phagocytosis achieves. Next, I will focus on the potential impact of developmental phagocytosis on the brain and discuss how these early life events may leave a lasting impact on microglia themselves. Finally, I will conclude by outlining outstanding questions that hold potential to uncover new insights into microglia function throughout the lifespan.

## 2 Phagocytosis shapes development across the brain in a diverse manner

Developmental phagocytosis of cells can be viewed as accomplishing one of two critical things: engulfing cells that are dying of programmed cell death (termed “efferocytosis” (Boada-Romero et al., 2020)) and engulfing what would otherwise be viable cells (termed “phagoptosis” (Brown and Neher, 2012; Vilalta and Brown, 2018)). The goal of efferocytosis is to maintain homeostasis by efficiently clearing apoptotic cells, whereas phagoptosis allows microglia to actively shape the cellular composition in a given brain region. In order to accomplish either goal, microglia detect chemoattractant “find me” signals (e.g. ATP and its derivatives, fractalkine, etc.) that are released by the target cell, and then sense membrane-bound “eat me” signals (e.g. phosphatidylserine, complement proteins, etc.) which triggers phagocytosis (Ravichandran, 2011). Alternatively, cells may express membrane-bound “don't eat me” signals (e.g. CD47, CD31, etc.), and it is the relative balance between “eat me” and “don't eat me” signals that ultimately determines whether a cell will be phagocytosed (Park and Kim, 2017). The mechanisms by which microglia find, recognize, and engulf their targets are numerous, and is a constantly evolving field of research; as such, I refer interested readers to comprehensive reviews on the subject (Medina and Ravichandran, 2016; Galloway et al., 2019; Lemke, 2019; Morioka et al., 2019).

Rodent studies indicate that microglial phagocytosis is a widespread phenomenon in the brain that varies dramatically in a region-, sex-, and age-dependent manner. For example, phagocytosis is highest at birth in the male rat amygdala while for other structures like the nucleus accumbens, phagocytosis increases equally in males and females over the first postnatal week (VanRyzin et al., 2019). There is an opposite sex difference just after birth in the rat hippocampus, as microglia are more phagocytic in newborn females than males (Nelson et al., 2017). In contrast to the amygdala and hippocampus, developmental phagocytosis occurs much later in the rat cerebellum, peaking in the third postnatal week (Perez-Pouchoulen et al., 2015, 2019)

The precise timing and degree to which phagocytosis occurs likely reflects the diverse maturation events taking place within each region (e.g. cell genesis, migration, death, etc.). Microglia regulate the cortical progenitor pool during embryonic development in rats and postnatal development in macaques by phagocytosing neural progenitors in the subventricular zone (Cunningham et al., 2013). Phagocytosis in the amygdala and hippocampus has been linked to sex differences in cell genesis during the postnatal period in rats, where microglia phagocytose astrocyte precursors and neural progenitors, respectively (Nelson et al., 2017; VanRyzin et al., 2019). Microglia within the hippocampus maintain a high phagocytic capacity throughout life, as they continue to regulate hippocampal neurogenesis into adulthood in mice (Sierra et al., 2010; Diaz-Aparicio et al., 2020). Microglia are not limited to engulfing neural progenitors, however; other identified targets include retinal ganglion cells in the embryonic mouse retina (Anderson et al., 2019) and oligodendrocyte progenitors in the developing mouse corpus callosum (Li et al., 2019; Nemes-Baran et al., 2020)

Together, the combination of region, timing, and substrate likely results in the diverse variety of functions developmental phagocytosis ultimately serves. For regions undergoing large-scale programmed cell death, developmental phagocytosis may serve as a homeostatic response to efficiently eliminate dead cells and prevent aberrant inflammatory reactions (Arandjelovic and Ravichandran, 2015). Tight coupling between apoptosis and phagocytosis results in rapid clearance of dying neural progenitors, for example, in the subgranular zone of the mouse hippocampus (Sierra et al., 2010). Similarly, a unique subset of microglia termed “proliferative-region-associated microglia” within the developing mouse corpus callosum phagocytose apoptotic oligodendrocytes (Li et al., 2019). When phagocytosis is disrupted by genetic deletion of specific phagocytic receptors (e.g. TREM2, CD11b, Mer, Axl) in mice, apoptotic cells accumulate through development (Takahashi et al., 2005; Wakselman et al., 2008; Fourgeaud et al., 2016).

In proliferative niches, phagocytosis may serve as a cellular pruning mechanism, simply culling the population of cells that are stressed, dying, or those that fail to properly integrate into circuitry. Initial evidence that microglia engulf viable cells during development was found in the rat cortex, where microglia phagocytose neural progenitors during late embryonic development. Pharmacological ablation of microglia increases the number of neural progenitors, indicating that microglia engulfment actively prunes the population (Cunningham et al., 2013). Microglia in the developing mouse retina engulf retinal ganglion cells (RGCs) as part of the normal reduction in RGC number. Disrupting phagocytosis by pharmacological inhibition or genetic ablation of the complement receptor 3 (CR3; also known as CD11b) increases the number of RGCs in the developing retina, without altering the dynamics of cell genesis or cell death (Anderson et al., 2019). Similarly, in the developing rat amygdala where microglia phagocytose newborn astrocytes, very few microglia phagocytic cups co-localize with cleaved caspase-3, a marker for apoptosis, suggesting that the engulfed cells are viable rather than dying.

Ultimately, developmental phagocytosis may be “directive” and essential for circuit formation and behavior expression. For example, I previously found microglial phagocytosis during rat amygdala development actively sculpts a sex difference in later-life social behavior. By engulfing more newborn astrocytes in males during the first postnatal week, juvenile males have fewer astrocytes in the medial amygdala than females, which inversely relates to a sex difference in rough and tumble play. The microglia-sculpted sex difference in astrocyte number likely regulates the sex difference in neuronal activation and play. When males are injected with a function-blocking antibody to prevent phagocytosis, newborn astrocyte engulfment is decreased, and the number of astrocytes is subsequently increased to female levels by the juvenile age. Blocking developmental phagocytosis alone has a drastic impact on neural activity and behavior, as the males now play at the lower female-typical levels and have female-typical immediate early gene expression (VanRyzin et al., 2019).

While the above highlights how phagocytosis impacts the brain, there is yet another interesting question that warrants discussion: what, if anything, does engulfing a cell (dead or otherwise) do to the microglia?

## 3 Do microglia undergo phagocytic reprogramming?

Does the influence of microglia stop once phagocytosis is complete, or does phagocytosis impart some more “lasting” changes to a microglial cell that feeds back into brain development? As has been demonstrated in the peripheral immune system, a phagocyte becomes functionally changed after ingesting a foreign substance like bacteria, for example. This process is described as “trained immunity” (Netea et al., 2020), the way in which the innate immune system “learns” about its environment and becomes fundamentally altered by it. So, might phagocytosis induce “training” of microglia immunity?

In fact, the entire process of phagocytosis—from “tasting” to “digesting”—can inform the phagocyte about the substrate being eaten. Information gained during each step of the phagocytic process is essential to generating a proper immune response (Underhill and Goodridge, 2012). Phagocytosis can reprogram microglia at the transcriptional, translational, and even epigenetic level (see Marquez-Ropero et al., 2020 for review). Thus, microglia may have a dynamic relationship with their “food” during development; microglia respond to local environmental cues to trigger phagocytosis, and in response, the substrate being phagocytosed likely feeds back information to the microglia.

In this sense, phagocytosis reprograms a macrophage and confers a “secondary” function. For example, efferocytosis or “silent killing” serves the primary purpose of removing apoptotic cells and secondarily programs the macrophage to stay in an un-inflamed state to preserve homeostasis (Boada-Romero et al., 2020). This is elegantly demonstrated using a parabiosis model in which the circulation of a wildtype non-fluorescent CD45.1 mouse is joined with a transgenic fluorescent (DsRed+) CD45.2 mouse. By using flow cytometry, the native macrophages from the CD45.1 mouse can be identified and analyzed for the presence of phagocytosed DsRed ​+ ​material. Transcriptomic analysis of DsRed+ and DsRed-macrophages from different tissue types demonstrates that while phagocytic and non-phagocytic macrophages share tissue-specific patterns of gene expression, those that have engaged in phagocytosis have an altered transcriptional profile that is downregulated for critical inflammatory pathways (A-Gonzalez et al., 2017).

While the idea of phagocytic reprogramming is only just gaining attention for microglia, recent studies suggest that a similar phenomenon may also occur in the brain. In the adult mouse hippocampus, microglia rapidly engulf apoptotic neurons to ensure homeostasis as described above (Sierra et al., 2010). This apoptosis-phagocytosis coupling achieves more than just dead cell removal, however, as apoptotic cell engulfment triggers immediate gene expression changes within the microglia. *In vitro* engulfment of apoptotic cells produces distinct gene expression profiles at both 3 ​h and 24 ​h, demonstrating that phagocytosis rapidly reprograms microglia and produces lasting alterations in their phenotype. While the short-term (3 ​h) changes in microglia phenotype are due to immediate regulation of transcription and translation, the long-term (24 ​h) effects may be attributable to epigenetic modifications as genes involved in chromatin remodeling and DNA metabolism were altered at this timepoint. As a result, this bidirectional communication between phagocytic microglia and their environment reprograms microglia to produce neurogenic modulatory factors, allowing microglia to maintain a proper balance between cell genesis and cell death (Diaz-Aparicio et al., 2020).

There is also evidence that epigenetic modifications underpin the unique phagocytic profile of microglia in the adult mouse cerebellum, as demonstrated in a recent study (Ayata et al., 2018). The cerebellum is one of a few brain structures that continues to lose cells during adulthood (Woodruff-Pak et al., 2010; Mortera and Herculano-Houzel, 2012), and microglia are essential to the rapid clearance of dying neurons in this region. As such, the transcriptional profile of cerebellar microglia is enriched for phagocytic activity and apoptosis-clearing genes, compared to microglia in the striatum where there is far less cell death (Mensah, 1982; Ayata et al., 2018). Interestingly, exposure of forebrain (i.e. less phagocytic) microglia to dying cells *in vitro* was sufficient to induce a pro-phagocytic gene profile similar to that seen in cerebellar microglia *in vivo*, without inducing a pro-inflammatory phenotype. Among the genes increased following exposure to apoptotic cells were the two histone demethylases, *Kdm6a* and *Kdm6b* (Ayata et al., 2018), which mediate the removal of the repressive H3K27me3 chromatin modification (Swigut and Wysocka, 2007). Moreover, the authors found that striatal microglia had significant enrichment of H3K27me3 at many of the cerebellar-expressed phagocytic genes, indicating that epigenetic modifications actively suppress a pro-phagocytic phenotype (Ayata et al., 2018).

The cerebellum is an interesting case study given the significant role microglia have in sculping the rodent cerebellum throughout development. Early in the postnatal period, microglia actively kill Purkinje cells via a superoxide burst to induce apoptosis as a method of ensuring proper cell number in mice (Marin-Teva et al., 2004), and microglia ablation during this time alters mouse Purkinje cell number and morphology (Kana et al., 2019). Later, during the third postnatal week in rats, microglia engulf apoptotic cells within the granule layer, presumably as a method of population refinement (Ashwell, 1990; Perez-Pouchoulen et al., 2015, Perez-Pouchoulen et al., 2019). These findings present the possibility that microglia acquire a pro-phagocytic phenotype early in development and retain such functional specialization throughout life via epigenetic means.

## 4 Outstanding questions and future directions

Despite these recent advances, many questions surrounding developmental phagocytosis remain unanswered. To what extent does microglial phagocytosis happen across the brain? Are all regions susceptible to microglia-mediated pruning throughout development? Or is there something unique about the regions that do undergo pruning? When and why is microglial phagocytosis most rampant within a given region, and does this reflect a critical period in development? What happens in contexts in which microglia phagocytic function is altered, such as perinatal infection, and how does this impact mature circuitry and behavior? At the most basic level, there is much still to do if we are to adequately understand how microglial phagocytosis shapes the developing brain.

Furthermore, these intriguing new findings highlight novel avenues of research: what are the consequences (if any) of developmental phagocytosis on microglia in the brain? Does phagocytosis feed back and instruct further microglia function to influence development? What reprogramming (if any) occurs as a result of a diverse microglia diet (i.e. engulfing neurons vs. astrocytes)? Might these early life events give rise to microglia regional heterogeneity and function later in life?

Microglia comprise a diverse and heterogeneous population during development (Li et al., 2019; Hammond et al., 2019), and further acquire region-specific phenotypes and function by adulthood (De Biase et al., 2017; Doorn et al., 2015; Grabert et al., 2016; Masuda et al., 2019). Given that microglia are relatively long-lived cells, and self-renew with consistent rates of turnover, it is an intriguing possibility that each cell will carry and pass along its “life history” long after the developmental period ends (Lawson et al., 1992; Askew et al., 2017; Ajami et al., 2007). In this way, microglia heterogeneity derived from different developmental niches could “imprint” later-life diversity via epigenetic modifications, similar to the concept of microglial priming due to early-life immune challenge (Bilbo and Schwarz, 2009).

Ultimately, it will be imperative to understand how these early-life events may impact microglia physiological states in adulthood or in conditions of pathology. Brain region-specific analyses and single-cell sequencing studies are revealing that microglia alterations as a result of biological sex (Hanamsagar et al., 2017; Guneykaya et al., 2018; Thion et al., 2018a), stress and aging (Tynan et al., 2010; Hart et al., 2012; Hickman et al., 2013; Kreisel et al., 2014; Grabert et al., 2016; Galatro et al., 2017), and neurodegenerative disease (Keren-Shaul et al., 2017; Krasemann et al., 2017; Mathys et al., 2017; Friedman et al., 2018) are quite heterogeneous. Whether or not phenotypic heterogeneity is driven by acute and diverse changes to the microenvironment, or if heterogeneity is “primed” earlier in life, remains to be established. Given the recent interest in understanding microglia heterogeneity in these contexts, I refer the reader to several recent reviews on the topic (Stratoulias et al., 2019; Masuda et al., 2020; Tan et al., 2020).

In conclusion, our understanding of the functional impact of microglial phagocytosis, particularly during development, has significantly advanced in recent years. However, there is still a wealth of information yet to be uncovered. A focus not only on how microglial phagocytosis shapes the brain, but also how phagocytosis shapes microglia, holds promises to reveal new dynamics by which microglia interact with and are changed by their local microenvironment.

## Declaration of competing interest

The author has no conflicts of interest to declare.
